# Papillary Thyroid Carcinoma Metastasis to the Epidural Space: A Case Report and Literature Review

**DOI:** 10.7759/cureus.78243

**Published:** 2025-01-30

**Authors:** Dia R Halalmeh, John Ochieng, Keilah Dos Santos, Yusuf-Zain Ansari, Marc D Moisi

**Affiliations:** 1 Neurosurgery, Hurley Medical Center, Flint, USA; 2 Neurosurgery, Michigan State University College of Human Medicine, Michigan, USA; 3 Neurosurgery, Temple University, Philadelphia, USA

**Keywords:** epidural metastasis, follicular thyroid carcinoma, papillary thyroid carcinoma, spinal metastasis, thyroid cancer

## Abstract

Papillary thyroid carcinoma is the most common thyroid cancer and commonly presents asymptomatically in a patient with cervical node enlargement. The defining characteristic of metastatic papillary thyroid carcinoma is its ability to spread through the lymphatic system. However, this case details a 71-year-old woman with metastatic papillary thyroid carcinoma who presents initially with complaints of back pain and leg weakness. Imaging showed an epidural mass with a pathologic fracture at the 10th thoracic vertebrae (T10). A T10 corpectomy with tumor excision and posterior T8-L1 laminectomy with fusion was performed, and the patient’s pathology report returned as a follicular variant of papillary thyroid carcinoma. The patient underwent a total thyroidectomy three months later. The literature review of similar cases was limited due to the rarity of this presentation, and our search returned only three previously reported cases of papillary thyroid carcinoma metastasizing to the epidural spine. Limited prognostic factors, including age and metastasis site, were found, which may be relevant to our patient’s outcomes. This case highlights the unique metastatic behavior of the follicular variant of papillary thyroid carcinoma and underscores its importance in expanding the current understanding of rare metastatic presentations. Furthermore, this case emphasizes the need for vigilance in diagnosing spinal metastases in patients with atypical presentations and demonstrates the importance of multidisciplinary management to achieve the best long-term outcomes.

## Introduction

Papillary thyroid carcinoma, one of the two differentiated variants of thyroid malignancies alongside follicular carcinoma, is characterized by specific nuclear features [1]. Representing roughly 80% of thyroid neoplasms, its incidence has increased from 4.8 to 14.9 cases per 100,000 individuals over approximately four decades, spanning from 1975 to 2013 [2]. The rising incidence is largely attributed to improved diagnostic modalities, including the widespread use of high-resolution ultrasonography and fine-needle aspiration biopsy, as well as heightened clinician awareness, all contributing to earlier detection of smaller, subclinical tumors [2]. Predominantly afflicting middle-aged adults, this disease demonstrates a notable prevalence among white females potentially influenced by genetic predispositions, hormonal factors, and environmental exposures that disproportionately affect this demographic group [3,4].

Papillary thyroid carcinoma typically manifests asymptomatically but is often accompanied by enlargement of cervical lymph nodes [2]. According to the American Thyroid Association guidelines, clinically apparent cervical lymph node metastases occur in up to 20-50% of patients at diagnosis [5]. Approximately 20% of cases exhibit signs of recurrent laryngeal nerve involvement, leading to vocal cord paralysis or tracheal compression, which manifests as hoarseness and dysphagia [2]. Another frequent initial finding on physical examination involves nodal metastases in the lateral neck, observed in approximately 27% of patients [6], highlighting the papillary carcinoma's propensity for lymphatic invasion. Despite these presentations, the overall prognosis remains favorable, with a 10-year survival rate ranging from 97% to 98% [7]. 

Although papillary thyroid carcinoma represents the predominant type of thyroid malignancy, metastasis to the thoracic spine is infrequent and even less common is the occurrence of metastatic involvement within the epidural space. The literature review revealed only three prior cases of epidural metastasis upon initial presentation [8-10]. Our case describes a patient presenting with non-radiating back pain as the primary manifestation of papillary thyroid carcinoma. Given the rarity of this clinical presentation, our case report outlining the management of this patient offers an intriguing contribution to the literature on this rare entity of epidural metastasis.

## Case presentation

Patient presentation

**Figure 1:**
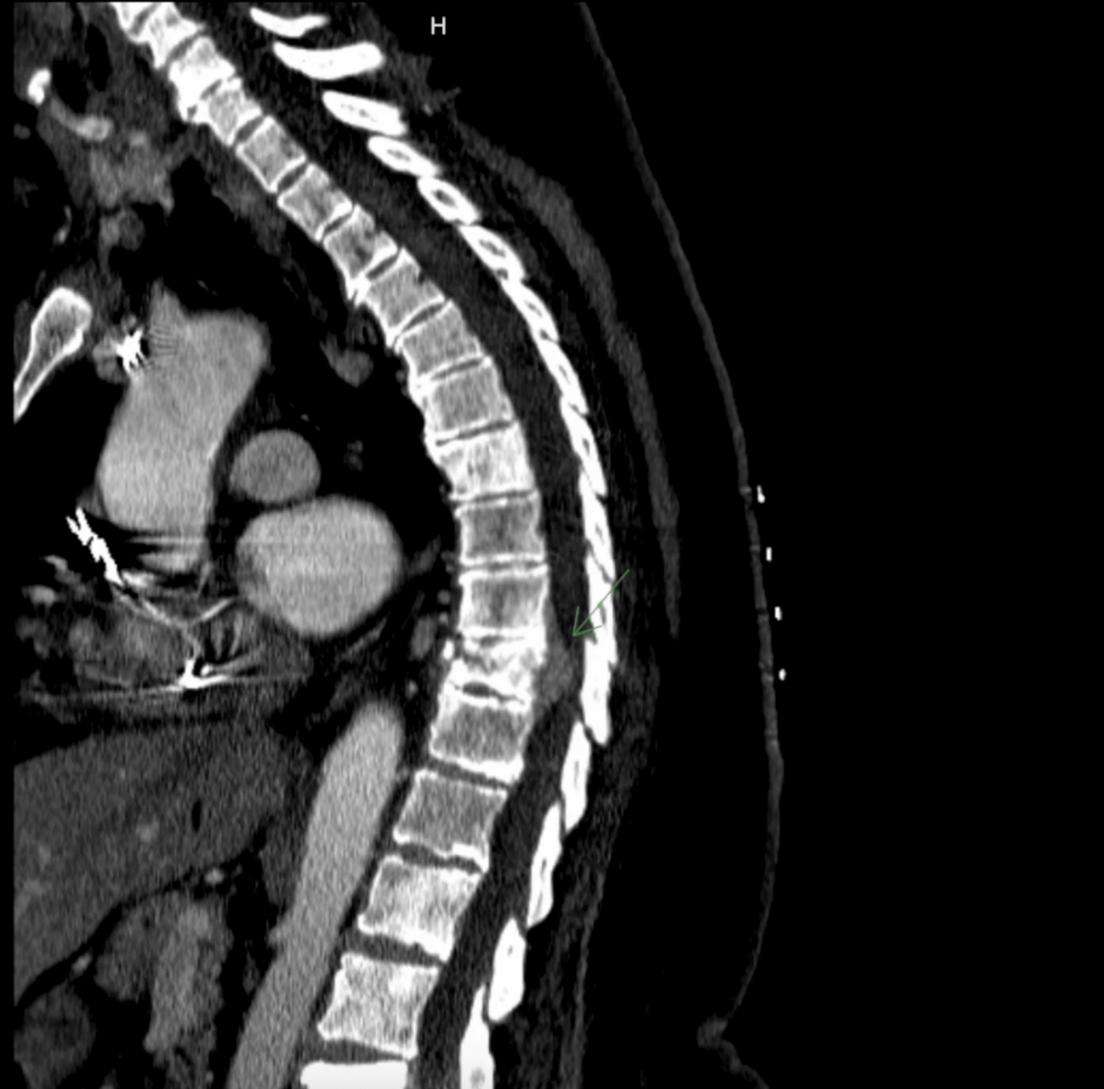
CT Scan Showing Intracanalicular Mass at T9-T11 With Fracture at T10

A 71-year-old African American female patient presented to the general surgery clinic with a chief complaint of non-radiating, burning back pain located in the lower thoracic region. Her medical history was significant for a spontaneous vertebral fracture for which she underwent a PET scan three months prior. She reported to have gone to an oncologist and underwent a PET scan in July, which showed a spinal fracture but was otherwise unremarkable. The patient denied any recent injuries, and a comprehensive neurological examination was negative for imbalance, stumbling, or changes in bowel and bladder habits. Physical examination was notable for weakness in the proximal muscles of the lower extremities bilaterally. Before this visit, the patient had undergone imaging, which included a thoracic CT with contrast in August 2023 and a dual-energy X-ray absorptiometry (DEXA) scan in September 2023. The CT scan (Figure 1) showed an intracanalicular mass at T9-T11 with a pathological fracture at T10. The DEXA scan noted markedly increased density of the L3 and L4 vertebra, indicating osseous metastatic disease.

Diagnostic workup

The relevant medical history of the patient includes hypertension, atrial fibrillation, obstructive sleep apnea, type 2 diabetes mellitus, and sick sinus syndrome. Relevant surgical history includes coronary angioplasty with stent placement, pacemaker implantation, joint replacement, and pacemaker generator exchange. The patient uses a cane to ambulate. There is no personal or family history of thyroid disorders, although there is a history of unspecified cancer in her father and two aunts, on both the maternal and paternal sides. The patient is a former smoker of one pack per day for 32 years and quit 22 years ago. Alcohol use includes three shots of liquor per week. The patient did not endorse any social concerns at the visit. 

Treatment and management

The patient was admitted to the neurosurgery department of a tertiary care center on January 15, 2024, for elective T10 corpectomy with tumor excision and posterior T8-L1 laminectomy with fusion. Prior to surgery, the patient was alert and oriented to person, place, and time. The patient stated the lower thoracic pain radiated intermittently to her flanks, following the T9 and T10 dermatome patterns. A neurological examination was repeated, denying any sensation of imbalance, paresthesias, numbness, or changes to her bowel and bladder habits. On a physical exam, the patient’s gait was found to be unsteady with continued weakness in her bilateral iliopsoas and quadriceps muscles. Muscle tone was found to be normal, and muscle strength was found to be 5/5 on all extremities except for bilateral iliopsoas and bilateral quadriceps, where muscle strength was 4/5. The intraoperative course consisted of a T9 to T11 laminectomy with costotransversectomy lateral extra cavitary approach with decompression of spinal nerve roots and resection of the intraspinal extradural mass and sent for diagnostic assessment. The operation was performed with no complications. 

Physical medicine and rehabilitation were consulted on January 16, 2024. The patient reported significant back pain but no new radiating pain. She also denied numbness and tingling in bilateral lower extremities. Physical medicine and rehabilitation determined there to be non-traumatic spinal cord injury with a thoracic spinal tumor and recommended full evaluation by rehabilitation services. It was anticipated that the patient would function at baseline and be able to return to her home. The patient was discharged on January 19, 2024, to a long-term acute care facility. The patient followed up with neurosurgery at two and four weeks, with reports of improving non-radiating back pain. At two weeks, the pain was still present around the incision site but had disappeared by the second follow-up appointment. Her pain regimen consisted of acetaminophen 1000 mg every eight hours. The patient also complained of continuing leg weakness at the first follow-up appointment. She presented to the office in a wheelchair and continued to work with physical therapy and occupational therapy at the long-term acute care facility. 

**Figure 2:**
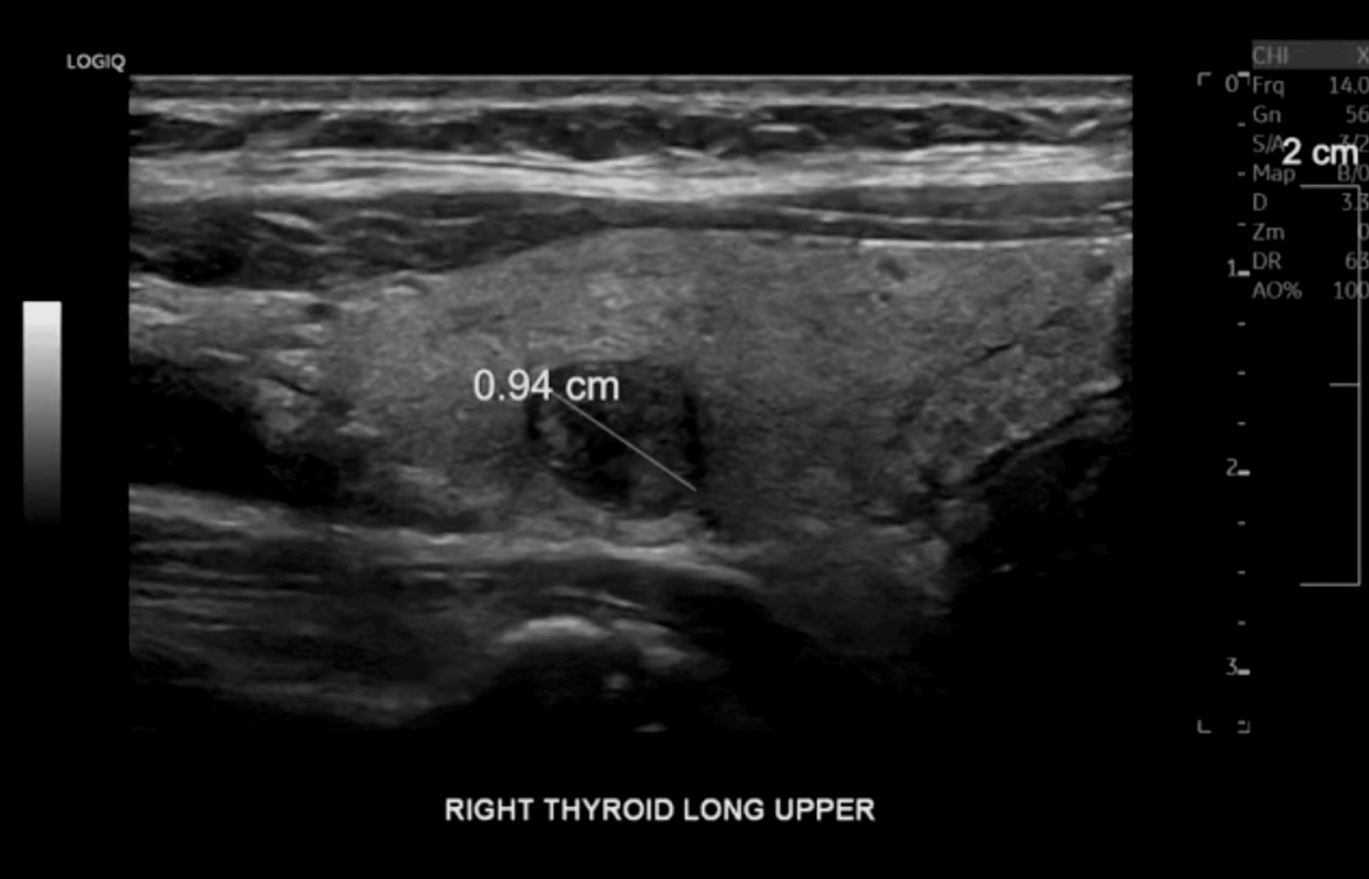
Ultrasound Imaging of Thyroid Nodule

Two soft tissue masses were removed during the surgery for biopsy. The final microscopic diagnosis from the specimens was metastatic well-differentiated thyroid carcinoma, favoring the follicular variant of papillary carcinoma. Due to the metastatic spread to the spine, the cancer was diagnosed as stage IV. Pending this pathology report, the patient was referred to oncology and endocrinology. The patient visited an endocrinologist in March 2024, and a thyroid ultrasound (Figure 2) was done in the office. The ultrasound showed a 0.9 cm thyroid nodule, TI-RADS 4, with an additional lymph node in the lateral neck compartment. Both the nodule and the lymph nodes were biopsied. Endocrinologist recommendations were total thyroidectomy and radiation oncology with high-dose radioactive iodine ablation given the metastatic nature of the cancer. 

At this point, the patient was scheduled to undergo a total thyroidectomy on April 3, 2024. The operation was performed with recurrent laryngeal nerve monitoring and no complications, and the final pathology report from this procedure was received on April 4th. The report read papillary carcinoma, an infiltrative follicular variant of the right thyroid lobe, measuring 0.9 cm x 0.8 cm in greatest measurement. Surgical margins were found to be clear. 

Following the total thyroidectomy, the patient remained in the hospital for two days. On postoperative day 1, the patient experienced mild oxygen desaturation during ambulation, necessitating 2 L/min oxygen supplementation via nasal cannula. Pulmonology assessed the condition as pulmonary edema secondary to fluid overload and possible respiratory infection. The respiratory panel was negative, and the patient was treated with a regimen of moxifloxacin and furosemide. Repeat chest X-rays confirmed improvement, and she was discharged with instructions for continued use of oxygen therapy and positive airway pressure support as needed.

## Discussion

Papillary thyroid cancer is the most common thyroid cancer, and it is known to metastasize primarily to lymph nodes, with distant metastasis being less common [11]. Additionally, it has been reported to metastasize to various locations including the lungs (48%), bone (18.3%), and liver (3.5%) [12]. Few reported cases of spinal metastasis have been published, emphasizing the importance of our patient’s case presentation, as it adds to the current literature. In 2021, Toriah reported on the metastasis and survival of papillary thyroid cancer, noting that the majority of metastasis was a single organ (74%). It is important to note that this case involves the follicular variant, which can exhibit distinct metastatic behavior compared to classic papillary thyroid cancer. The distinction between single and multiple organ distance metastasis has been reported to have an impact on overall survival. Toriah further noted that patients with a single metastatic site presented with the lung as a preferential site followed by bone metastasis. This is in comparison to concomitant metastasis to the brain and lung or brain and liver [12]. 

Metastasis of papillary thyroid carcinoma to the bone has been documented to present with a variety of initial symptoms consisting of pain, motor, autonomic, or sensory dysfunction-dependent bone involvement in addition to other factors [13]. Pain is the most common symptom, occurring in 83-95% of patients, and can precede the progression of other neurologic symptoms [13]. There are three types of pain that affect symptomatic patients, local, mechanical, and radicular pain. Following pain, motor dysfunction is the next most common symptom in patients with metastatic disease. Weakness of one or more muscle groups has been reported in 60-85% of patients with metastatic epidural spinal cord compression [13]. In a retrospective analysis of 202 thyroid cancer patients with spinal metastasis, 57% of patients presented with symptoms of nerve compression. 45% of patients had spinal cord compression and 12% with root compression [14]. 

Treatment of metastatic papillary thyroid carcinoma to the spine involves the collaboration of multiple specialties, including surgical, medical, and radiation oncology, interventional radiology, rehabilitation, and pain specialists. Our patient initially presented to general surgery and subsequently saw neurosurgery. Other specialists involved in her care included oncology, pathology, ENT, rehabilitation, pulmonology, and medicine. Literature has reported initial treatment of most thyroid cancers is a surgical resection followed by a biopsy to determine histology [15]. However, in well-differentiated thyroid cancer with bone metastasis, radioiodine therapy can be advantageous for 50% of patients who have shown complete response to iodine treatment [16]. The second line therapy for spinal metastasis is total en bloc spondylectomy when there are indications that lesions may have extension with spinal cord compression and neurological deficit or compression fracture [16]. If unresolved, third-line therapy includes external radiation, kyphoplasty, stabilization, radiosurgery, or ablation techniques with adjunctive therapy being denosumab [16].

There are only a few reported cases of epidural lesions secondary to metastatic papillary thyroid carcinoma in the literature (Table 1). Goldstein et al. reported the case of a 58-year-old man with no previous symptoms of thyroid abnormality who began experiencing back pain and later developed neurological symptoms indicative of spinal cord compression [5]. Radiographic evaluations revealed a large osteolytic lesion of the T10 vertebral body, which was found to be a metastasis from a thyroid carcinoma following further investigation including ultrasound, CT, and eventually thyroidectomy. Histologically, the metastatic lesion exhibited a papillary pattern typical of well-differentiated papillary thyroid carcinoma. Management included decompressive laminectomy followed by total thyroidectomy and radioactive iodine therapy, which aims to reduce tumor load and prevent further metastatic spread [5].

**Table 1:** Epidural Lesions Secondary to Papillary Carcinoma Metastasis Reported in Literature

Reference	Case	Age/sex	Location	Presentation	Treatment/outcome
Goldstein et al., 1988 [5]	Case #1	58-year-old male	Metastasis to T10	Back pain, paraplegia	Improved after decompressive laminectomy and radioiodine therapy
Zhang et al., 2013 [6]	Case #2	63-year-old female	Cervical spine (C4-C5)	Cervical pain and neurologic deficit	Improved after surgery
Zhang et al., 2013 [6]	Case #3	45-year-old male	Cervical spine (C1, C2, C5)	Swallowing difficulties and a hoarse voice	Improved post-surgery
Zhang et al., 2013 [6]	Case #4	65-year-old female	Cervical spine (C2)	Cervical pain and neurologic deficit	Underwent surgery
Zhang et al., 2013 [6]	Case #5	72-year-old female	Cervical spine (C5-C6)	Pain, neurological deficit for 2 years	Improved after surgery
Zhang et al., 2013 [6]	Case #6	57-year-old female	Thoracic spine (T9)	Pain, neurological deficits	Improved after surgical intervention
Zhang et al., 2013 [6]	Case #7	78-year-old male	Thoracic spine (T2)	Back pain and neurological deficits for 3 months	Improved following surgery
Saad et al., 2022 [7]	Case #8	65-year-old female	Thoracic spine (T1-T3)	Paraplegia	Underwent successful decompressive surgery

A retrospective study by Zhang et al. details the clinical presentations, treatments, and outcomes of patients with epidural spinal cord compression due to metastatic thyroid cancer [6]. From this study, there are six cases of papillary thyroid carcinoma metastatic to the epidural space. Specific details from the study show that patients with papillary thyroid carcinoma metastases to the epidural space generally have a good prognosis when aggressive surgical resection is performed. The study emphasizes that such aggressive surgical approaches can lead to significant improvements in neurological function and pain relief. The patients also received adjuvant therapies such as bisphosphonate treatment post-surgery to help control skeletal-related events. It is noteworthy that in these cases, the duration of preoperative symptoms ranged from one to 24 months, with an average of approximately six months [6]. 

Saad et al. present the case of a 65-year-old African American female with a history of papillary thyroid carcinoma under radiotherapy treatment who exhibited rapidly progressive bilateral lower extremities weakness due to metastatic thyroid carcinoma causing destructive epidural lesions at T1-T3 vertebrae with thoracic cord compression [7]. The patient's symptoms included weakness, loss of sensation associated with upper back pain, and no bowel or bladder incontinence. The patient underwent emergency decompressive laminectomy and debulking of the lesions. The histopathological examination of the surgical biopsy confirmed the metastatic papillary thyroid carcinoma of the follicular variant, which was consistent with the original thyroid tumor’s biopsy. Postoperatively, the patient had a successful neurological recovery with physiotherapy and was scheduled for completion of radiotherapy. She was discharged into a rehabilitation facility for ongoing care. A whole-body I-131 scan performed post-radiotherapy revealed uptake in the thyroid bed with retrosternal tissue extension, but no evidence of osseous metastasis was found [7].

Due to the limitation in the number of reported cases with metastatic papillary thyroid carcinoma presenting with spinal compression, it is difficult to comment on prognostic factors to predict patient outcomes. Nonetheless, some comparisons made in the literature between papillary thyroid carcinoma and follicular thyroid carcinoma may inform the prognostic outlook in relevance to our patient. Although papillary thyroid carcinoma accounts for the majority of thyroid cancer, it is less likely than follicular thyroid carcinoma to metastasize to the spine [6]. Furthermore, patients with papillary thyroid carcinoma generally have a better survival rate in comparison to patients with follicular thyroid carcinoma, but the presence of spinal metastasis results in a worse prognosis for patients with papillary thyroid carcinoma [6]. Literature also suggests that age at diagnosis may be an independent prognostic factor for the overall survival of thyroid cancers [5,6]. In a case series of 46 patients with either metastatic follicular or metastatic papillary thyroid carcinoma, it was found that older patients (greater than age 40) with follicular carcinoma were more responsive to radioiodine therapy than similarly aged patients with papillary thyroid carcinoma [5]. To put it in perspective, the five-year survival rate was 100% in those with responsive radioiodine therapy to follicular thyroid carcinoma, whereas the five-year survival rate was 14% in the patients with responsive radioiodine therapy to papillary thyroid carcinoma [5].

## Conclusions

The defining characteristic of papillary thyroid carcinoma is its ability to metastasize through the lymphatic system, with the follicular variant demonstrating a higher propensity for distant metastasis, including to the spine. This histologic distinction is critical in understanding the metastatic behavior observed in this case. The presentation of metastatic papillary thyroid carcinoma to the epidural space of the thoracic spine is exceedingly rare and represents an important addition to the literature. Due to its rarity, the scope was limited in terms of finding evidence to inform the treatment of this disease presentation. In this case, a multidisciplinary approach was instrumental in managing the complex presentation, combining neurosurgery, oncology, endocrinology, and rehabilitation services to address the patient’s needs holistically. Limited evidence highlights that factors such as age at diagnosis and site of metastasis are key prognostic indicators that should be taken into account when determining patient outcomes. It is the hope that more research into this unique disease presentation will provide more definitive data and benefit patients with similar conditions in the future.
